# Supplementary material for: Natural Variations in Key Maturity Genes Underpin Soybean Cultivars Adaptation Beyond 50° N in Northeast China
Source: Int J Mol Sci. 2025 Apr 3;26(7):3362. doi: 10.3390/ijms26073362 (PMC11989263; doi:10.3390/ijms26073362)
Supplement: Supplementary file 1 [file ijms-26-03362-s001.zip › Supplementary Figures.pdf]

International Journal of *Molecular Sciences*

MDPI

Supplementary Materials

Natural Variations in Key Maturity Genes Underpin Soybean Cultivars Adaptation Beyond 50° N in Northeast China

Hongchang Jia [1,2,3], Baiquan Sun [2,4], Bingjun Jiang [2], Peiguo Wang [1,2,4], Mahmoud Naser [2], Shuqing Qian [2], Liwei Wang [2,4], Lixin Zhang [2], Mikhail Sinegovskii [5], Shi Sun [2], Wencheng Lu [3], Valentina Sinegovskaya [5], Jiangping Bai [1,*] and Tianfu Han [1,2,4,*]

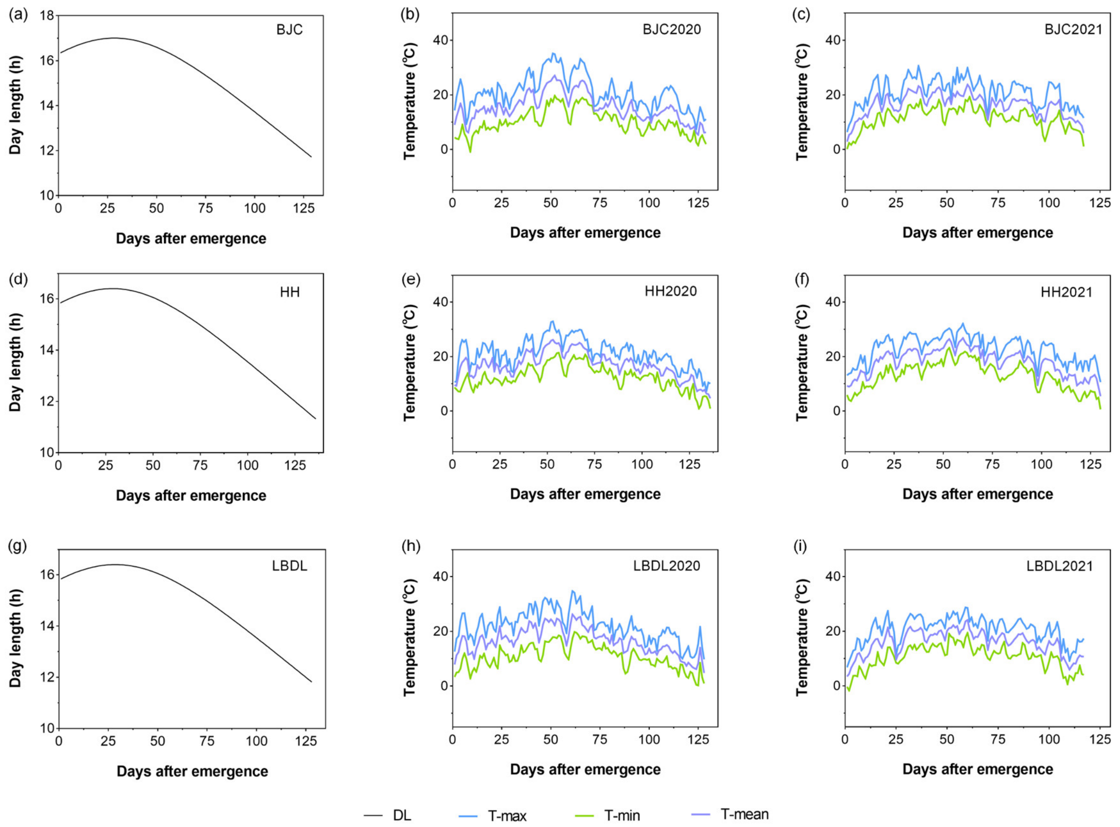

Figure S1. Daily mean (T-mean), maximum (T-max), and minimum (T-min) temperatures, and day length (DL) from the planting dates until the first frost dates at three experimental locations. Data for 2020 and 2021 are shown for Beijicun (BJC), Heilongjiang, China; Heihe (HH), Heilongjiang, China; and Labudalin (LBDL), Inner Mongolia, China.

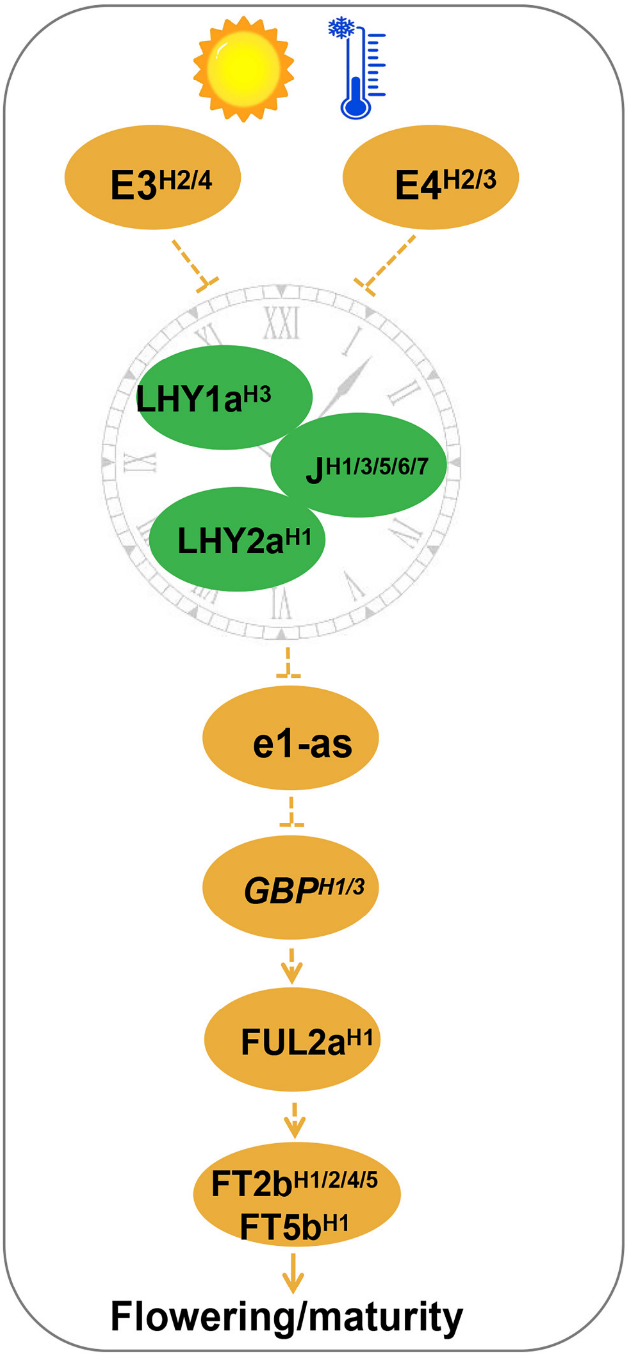

Figure S2. Dominant haplotype combinations and molecular regulatory network of key genes for photothermal responses in soybean cultivars from regions north of 50°N latitudes. Arrows indicate transcriptional activation, while perpendicular bars represent transcriptional repression.
